# Potential wound healing activity of *Quercus infectoria* formulation in diabetic rats

**DOI:** 10.7717/peerj.3608

**Published:** 2017-07-24

**Authors:** Julalak Chokpaisarn, Sasitorn Chusri, Thanaporn Amnuaikit, Wandee Udomuksorn, Supayang Piyawan Voravuthikunchai

**Affiliations:** 1 Department of Microbiology and Excellence Research Laboratory of Natural Products, Faculty of Science and Natural Product Research Center of Excellence, Prince of Songkla University, Hat Yai, Songkhla, Thailand; 2 Faculty of Traditional Thai Medicine and Excellence Research Laboratory on Natural Products, Faculty of Science and Natural Product Research Center of Excellence, Prince of Songkla University, Hat Yai, Songkhla, Thailand; 3 Department of Pharmaceutical Technology and Excellence Research Laboratory on Natural Products, Faculty of Science and Natural Product Research Center of Excellence, Faculty of Pharmaceutical Sciences, Prince of Songkla University, Hat Yai, Songkhla, Thailand; 4 Department of Pharmacology, Faculty of Science, Prince of Songkla University, Hat Yai, Songkhla, Thailand

**Keywords:** Diabetic wounds, *Quercus infectoria*, Wound healing process, Wound treatment

## Abstract

**Background:**

*Quercus infectoria* G. Olivier (Fagaceae) nutgalls have been widely employed in traditional Asian medicine for several treatments, especially wounds and skin disorders. However, the effects of this plant on wound healing have not yet been clearly elucidated. This present work was focused on utilization of *Quercus infectoria* (Qi) as a topical agent for chronic wound treatment.

**Methods:**

Twenty Qi formulations (QiFs) were pharmaceutically formulated and antibacterial activity of all formulations was performed. The best formulation based on an antibacterial activity was selected for evaluation of wound healing property. Total phenolics, total flavonoids, and an anti-oxidant activity of the selected formulation were also investigated. Wound healing activity was assessed in streptozotocin-induced diabetic rats and control rats. Streptozotocin injection (50 mg/kg) was found to induce marked hyperglycaemia, compared with citrate-injected controls. Two wounds were created on the upper back of each animal. QiF was topically applied three days after wounding to one of the duplicate wounds on each animal and physiological saline (control) was applied to the other. All wounds were cleaned once a day until wound closure.

**Results:**

QiF10, which exhibited antibacterial and anti-oxidant activities, had the ability to enhance the wound healing process in diabetic rats with abundant cellular infiltration, collagen deposition, and re-epithelialization when compared with the control.

**Discussion:**

This study suggested that QiF10 could be a novel alternative treatment for diabetic wounds.

## Introduction

Diabetic ulcers, one of the most common prevalent complications in diabetic patients, are becoming a serious concern worldwide. According to the International Diabetes Federation, around 415 million people worldwide were estimated to have diabetes in 2015, and this will increase up to 642 million people by 2040 (International Diabetes Federation, 2015). They are considered as a high risk factor for lower-limb amputation, representing a main waste of cost and healthcare resources. Every year, more than a million lower limbs of diabetic patients are amputated as a consequence of wound advancement (Bakker & Schaper, 2012). Development and progression of diabetic ulcers are complicated and are different from other chronic wounds that involve the convergence of several pathological mechanisms such as neuropathy, foot deformity, vascular diseases, as well as infections (Guo & DiPietro, 2010).

Treatment of diabetic wounds requires multidisciplinary strategies based on the underlying diseases, infections, and wound environment. Conventional care practices (including topical antibiotics, dressing containing antibiotics, antiseptic agents, and even debridement) are commercially used to control infections and to promote the wound healing process (Wound International, 2013). However, they are always insufficient and some wounds do not respond to these therapies, worsening wound healing management (Kim & Steinberg, 2012; Trivedi et al., 2014). Nowadays, the occurrence of multidrug-resistant organisms has emerged as a serious and common concern in diabetic wounds, leading to a longer hospital stay duration, increased economic burden, morbidity, and in some cases may lead to higher mortality (Trivedi et al., 2014). Therefore, development of a new appropriate therapeutic approach for the treatment of diabetic wounds is urgently needed.

During the last decades, medicinal plants, an important source of phytochemical compounds, are of interest for treatment of several diseases. The nutgall of *Quercus infectoria* G. Olivier (Fagaceae), a pathological excrescence formed on branches of the plant, has been used since 1961 as an astringent agent (Sariozlu & Kivanc, 2011). In traditional Asian medicine, it has been extensively applied for treatment of infectious diseases, skin disorders, and inflammatory ailments (Sariozlu & Kivanc, 2011). Several scientific researches have demonstrated the biological properties of the plant, confirming the traditional use for broad spectrum antibacterial (Voravuthikunchai, Chusri & Suwalak, 2008), anti-inflammatory (Kaur et al., 2004; Chokpaisarn et al., 2017) and anti-oxidant activities (Kaur, Athar & Alam, 2008). Our previous reports revealed that the ethanolic extract of nutgall displayed a competent antibacterial ability against most important skin pathogens causing wound infections and can also interfere with the formation of staphylococcal biofilm (Voravuthikunchai, Chusri & Suwalak, 2008; Chusri, Phatthalung & Voravuthikunchai, 2012).

Based on its medicinal use and biological efficacy, nutgall extract is considered to be an essential source for utilization as a topical agent for wound treatment. In the present study, the ethanolic extract of nutgalls was applied in a dosage form of solution for chronic wound treatment. The effects of *Quercus infectoria* formulation (QiF) on the wound healing process were investigated using diabetic and non-diabetic rat models.

## Materials & Methods

### Preparation of extract and topical agent

Nutgalls from *Quercus infectoria* were purchased from Thai Herbal shop, Hat Yai, Songkhla, Thailand. Botanical identification of the material was obtained by a special botanist. A voucher specimen of them is collected at Natural Research Center of Excellence, Prince of Songkla University. The powder of nutgalls was extracted as previously described (Chusri & Voravuthikunchai, 2009). For pharmaceutical formulation, the extract was dissolved in 95% ethanol or sterile distilled water at concentrations of 15–40% (w/v) and tested for antibacterial activity. Based on antibacterial activity and solubility, the extract at concentration 30% (w/v) was used for further formulation. Twenty formulations were prepared from the extract and various concentrations of propylene glycol, polyethylene glycol 400 (PEG-400), polysorbate 20, polysorbate 60, 95% ethanol, and uniphen P-23 (Table 1). The volume of each formulation was made up to 100 mL with sterile distilled-deionized water. Each formulation was stored in a sterile bottle at room temperature until use.

**Table 1:** Composition, minimum inhibitory concentration (MIC) values of Qi formulations against *Staphylococcus aureus* ATCC 25923 and methicillin-resistant *S. aureus* NPRC R001.

Formulas^a^	Qi (g)	Ingredients (%, w/v)							MIC values (mg/mL)	
		Propolene glycol	95% Ethanol	PEG-400	Polysorbate 60	Polysorbate 20	Uniphen P-23	dH_2_O	*S. aureus* ATCC 25923	MRSA
QiF01	30	30	10	15	5	5	1	34	9.38	4.69
QiF02	30	30	4	25	4	4	1	32	2.34	4.69
QiF03	30	25	10	15	5	5	1	39	18.75	9.38
QiF04	30	25	2	20	4	4	1	44	18.75	4.69
QiF05	30	25	15	15	3	3	1	38	9.38	4.69
QiF06	30	20	10	20	3	2	1	44	18.75	4.69
QiF08	30	20	5	25	5	5	1	39	18.75	9.38
QiF09	30	20	10	20	2	8	1	39	37.5	9.38
QiF10	30	20	5	25	3	2	1	44	0.29	0.29
QiF11	30	20	4	25	3	3	1	44	18.75	9.38
Base formulations^b^	0								–^c^	–

**Notes.**

a Twenty of Qi formulations (QiFs) were pharmaceutically formulated but QiF7, QiF12-20 were not presented because they were not soluble in the bacterial media.

b Base formulations of QiF01–QiF20 contains all ingredients except Qi extract.

c –= no activity.

### Antibacterial activity

Broth microdilution method was performed to determine the minimal inhibitory concentration (MIC) of 20 Qi formulations against *Staphylococcus aureus* ATCC 25923 and methicillin-resistant *S. aureus* (MRSA) NPRC R001. The bacterial pathogens were cultured in Mueller-Hinton broth (MHB, Difco, France) at 37 °C for 4 h. The suspensions were adjusted to McFarland standard No. 0.5 and diluted with MHB to reach a cell density corresponding to 10^6^ CFU/mL. Qi formulations were diluted using two-fold dilution method. The bacterial suspension was then added with 100 µL of two-fold serially diluted of each Qi formulation in a sterile 96-well microtiter plate. After 18 h of incubation, the bacterial growth was measured using a microplate reader (Sunrise, Tecan, Switzerland) at 620 nm. The MIC is defined as the lowest concentration that produces a complete inhibition of bacterial growth. An experiment was done in triplicate. Bacterial culture with MHB without the addition of each Qi formulation was used as a control. The most active formulation with low MIC value was selected for further experiment.

### Phytochemical screening

Total phenolic compounds of both the selected formula and the extract were assessed using the Folin-Ciocalteu agent (Fattahi et al., 2014). The diluted solution (100 µL) at concentration 100 µg/mL was mixed with 750 µL of 10% Folin-Ciocalteu’s reagent dissolved in sterile distilled deionized water. After incubation, 750 µL of 6% (w/v) sodium bicarbonate was added and further incubated for 30 min in dark at room temperature. The absorbance was measured at 760 nm using UV-visible spectrophotometer (Thermo Fisher, Waltham, MA, USA). Quantification was done on a standard curve of gallic acid (Sigma, Germany). The results were expressed as gallic acid equivalents (mg of GA/g of extract).

The aluminium chloride colorimetric assay was performed to determine total flavonoid content (Fattahi et al., 2014). Each tested sample (1 mL) was added into test tubes along with 4 mL of distilled deionized water and 0.3 mL of 5% sodium nitrite solution. After 5 min of incubation, 0.3 mL of 10% aluminium chloride was added and further incubated for 6 min at room temperature. Then, 2 mL of 1 M sodium hydroxide was added and the volume of the mixture was making up to 10 mL with distilled water. The mixture was carefully mixed before determination. The absorbance was measured at 510 nm using UV-visible spectrophotometer. The blank was performed using distilled water. Catechin was used as a standard control. The samples were performed in triplicates. The data of total flavonoid were expressed as mg of catechin equivalents/100 g of dry extract.

### Reverse phase high performance liquid chromatography (RP-HPLC) analysis

The quality of the selected formulation was analysed by HPLC using High Performance Liquid Chromatograph 1100 (Aligent Technologies, Santa Clara, CA, USA) according to the previous report (Pin et al., 2006). Gallic acid (Sigma, Darmstadt, Germany) was used as a chemical marker. The analytical column used was Hypersil ODS 4.0 × 250 mm with packing material of 5µm particle size at a flow rate of 1 mL/min at room temperature. The mobile phase consisted of 0.1% phosphoric acid (solvent A) and acetonitrile (solvent B). The gradient elution employed was as follows: 85%A at 0–12 min, 75%A at 12–20 min, 85%A at 0–22 min, 85%A at 22–25 min. Each injection contained 10 µL of the sample. Detection was monitored at wavelength 280 nm.

### Anti-oxidant activity

An anti-oxidant activity of both the selected formulation and the extract was performed by DPPH and ABTS scavenging assay (Meda et al., 2005; Thaipong et al., 2006). For DPPH scavenging assay, the tested formulation and the extract were dissolved in methanol and mixed with 1.5 mL of 80 µM DPPH solution in methanol. After 15 min of incubation in the dark, the absorbance of the reaction was determined at 517 nm UV–V is spectrophotometer.

For ABTS scavenging assay, an aliquot solution of 7 mM ABTS (Merck, Germany) was oxidized with potassium persulfate for 16 h in the dark at room temperature. The ABTS+solution was diluted with phosphate buffer saline (PBS; Merck, Darmstadt, Germany) pH 7.4 to initial an absorbance of 0.70 ± 0.02 at 734 nm. An aliquot (20 µL) of each formulation was mixed with 2 mL ABTS+solution for 6 min in the dark. The absorbance was read at 734 nm using UV-visible spectrophotometer. Trolox was used as a positive control for both experiments. The radical scavenging activity was calculated according to the following formula;

\begin{eqnarray*}\text{%}Inhibition=[(blank absorbance-sample absorbance)/blank absorbance]\times 100 \end{eqnarray*}

### Stability test

Stability of the most active Qi formulation (QiF10) at different temperatures was determined using stability test. The sample was kept under two different temperatures at 4 °C for 24 h and 60 °C for 24 h, three cycles. After incubation, the sample was observed for changes in appearance, such as colour, precipitation, separation, and pH (Reddy & Murthy, 2005).

### Animals

Male wistar rats (7–8 weeks, 200–250 g) were acclimated to standard housing and fed under a protocol approved by the Animal Ethic Committee, Prince of Songkla University, Thailand (Ref.12/2014). All animals were housed in individual cages under constant temperature (22 °C) and humidity with a 12-hour light-dark cycle.

### Diabetic rat model

Rats were divided into two groups: diabetes (*n* = 16) and non-diabetes (*n* = 16). All animals were fasted overnight. Diabetes was induced by intraperitoneal injection of freshly prepared solution of streptozotocin (50 mg/kg) in 0.1 M citrate buffer (pH 4.5). Non-diabetic rats were injected with citrate buffer alone. After seven days of injection, blood was taken from the tail to measure blood sugar levels by glucometer. The animals were considered as diabetes, if their blood sugar level was 250 mg/dL or higher (Ravi, Ramachandran & Subramanian, 2004).

### Wound protocol

For wounding, the animals were anaesthetized with intraperitoneal injection of thiopental sodium (55 mg/kg). The dorsal area was completely shaved and sterilized with 70% ethanol. In addition, 2% lidocaine was applied as a topical anaesthesia. Treatment and control were performed in the same animal in order to minimise bias. Two 1-cm^2^ excision wounds were created on the upper back of each animal with a scalpel. Wounds were left to chronic state for three days post-wounding. On day 4, 15 µL of the selected Qi formulation (30% w/v) was topically applied to one of duplicate wounds on each animal and physiological saline (control) was applied to the other. Each treatment was given daily until wound closure. On day 0, 3, 7 and 14 post-treatment, wounds (*n* = 8) in each animal group were photographed and the wound size was measured by computing software ImageJ. Data were reported as percentage wound closure calculated by the following formula;

\begin{eqnarray*}\text{%}wound~closure=({A}_{0}-{A}_{1}/{A}_{0})\times 100 \end{eqnarray*}

*A*_0_ = Wound area on day 0

*A*_1_ = Wound area on day 3, 7, or 14 post-treatment

### Histological analysis

On day 0, 7, and 14 post-treatment, animals were sacrificed and wound samples (*n* = 4) from each group were collected for histological analysis. Tissues were fixed in formalin and embedded in paraffin. Six-micrometer paraffin sections were stained with hematoxylin and eosin (H&E) for measurement of re-epithelialization and granulation tissue production under a light microscope. The images were photographed.

### Statistical analysis

All tested groups were compared with the control groups and the results were analysed statistically using *t*-test. Continuous variables were described in terms of mean and Standard Error of Measurement (SEM). All calculations were performed by SPSS 14.0 (SPSS Inc., Chicago, IL, USA). The values of *p* < 0.05 were considered to be statistical significance.

## Results

### Selection of formulation and antibacterial activity

An antibacterial activity of 20 Qi formulations was tested to determine MIC values against MRSA. Table 1 displays composition and antibacterial activity of 10 representative Qi formulations. The others were not presented because the extract was not completely soluble for antibacterial assay. MIC range was found between 0.29 to 37.5 mg/mL. It was observed that QiF10 exhibited antibacterial activity against MRSA with MIC values at 0.29 mg/mL. Thus, the formulation of QiF10 was selected for further experiment. The base solution of all formulations did not show an antibacterial activity (Table 1). The stability of QiF10 was determined by observing the formula appearance, including colour, odour, precipitation, and pH value. There was no change in all observed characteristics (Table S1).

### Phytochemical screening and HPLC

**Figure 1:**
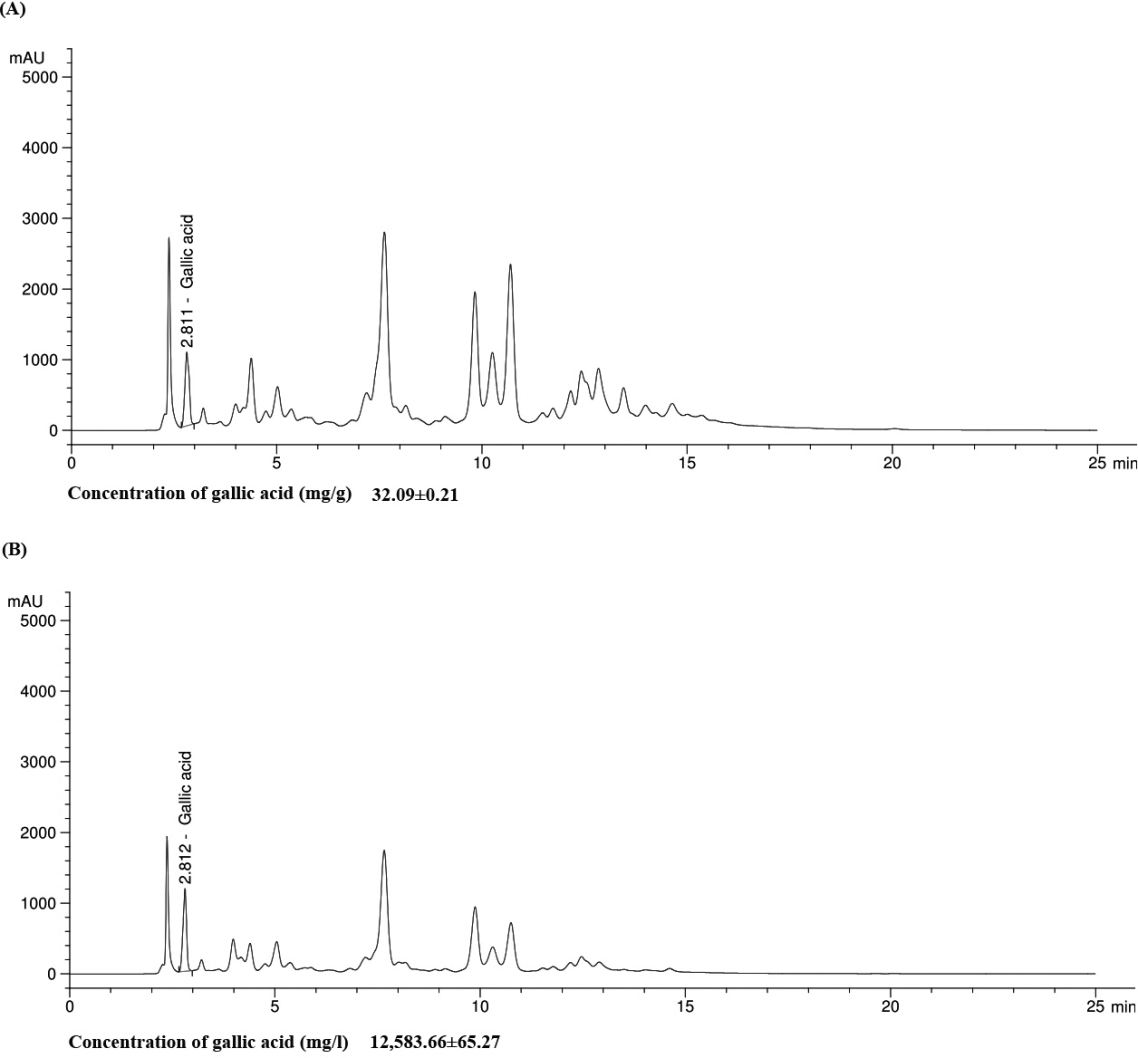
Chromatogram of gallic acid in both Qi extract (A) and QiF10 (B).

Total phenolic and total flavonoid compounds were determined. The results demonstrated that the ethanolic extract consisted of high concentration of total phenolic compounds (954.07 mg GAE/100 g) which was close to standard gallic acid (960.85 mg GAE/100 g), while QiF10 had total phenolic compound at 580.34 mg GAE/100 g (Table 2). Both the extract and the formulation showed less total flavonoid contents than the standard control. There were 247.22 mg CE/100 g and 101.39 mg CE/100 g for the extract and the formulation, respectively (Table 2). Furthermore, HPLC analysis of gallic acid in the extract and the formulation were 32.09 mg/g and 12,583.66 mg/L, respectively. The chromatograms of gallic acid quantified in of the extract and QiF10 were presented in Fig. 1.

**Table 2:** Total phenolics, total flavonoids, and an anti-oxidant activity of QiF10 and Qi extract.

Samples	Phytochemical screening		Anti-oxidant activity (% inhibition)	
	Total phenolics (mg GAE/100 g ± SD)	Total flavonoids (mg CE/100 g ± SD)	DPPH (mean ± SD)	ABTS (mean ± SD)
Qi extract	954.07 ± 8.39	240.28 ± 43.11	97.40 ± 0.001	98.70 ± 0.004
QiF10	580.34 ± 4.79	115.28 ± 24.07	94.47 ± 0.001	96.43 ± 0.003
QiF10 base formulation	0	0	–^a^	–
Trolox	NA^b^	NA	97.65 ± 0.001	99.15 ± 0.001
Gallic acid	960.85 ± 56.33	NA	NA	NA
Catechin	NA	823.61 ± 127.29	NA	NA

**Notes.**

a –= No activity.

b NA = Not Applicable.

### Anti-oxidant activity

DPPH and ABTS radical scavenging ability of QiF10 and the extract are shown in Table 2. High percentage of DPPH and ABTS inhibition was noticedin the extract testing group with percentage inhibition at 97.40% and 98.70%, respectively. The formulation also exhibited a high percentage of DPPH and ABTS inhibition with 94.48% and 96.43%, respectively. Interestingly, these inhibition values were very close to standard control trolox which were 97.62% and 99.14% against DPPH and ABTS, respectively (Table 2). An anti-oxidant activity did not observe in the base formulation of QiF10 (Table 2).

### Streptozotocin-induced diabetic condition in rats

**Figure 2:**
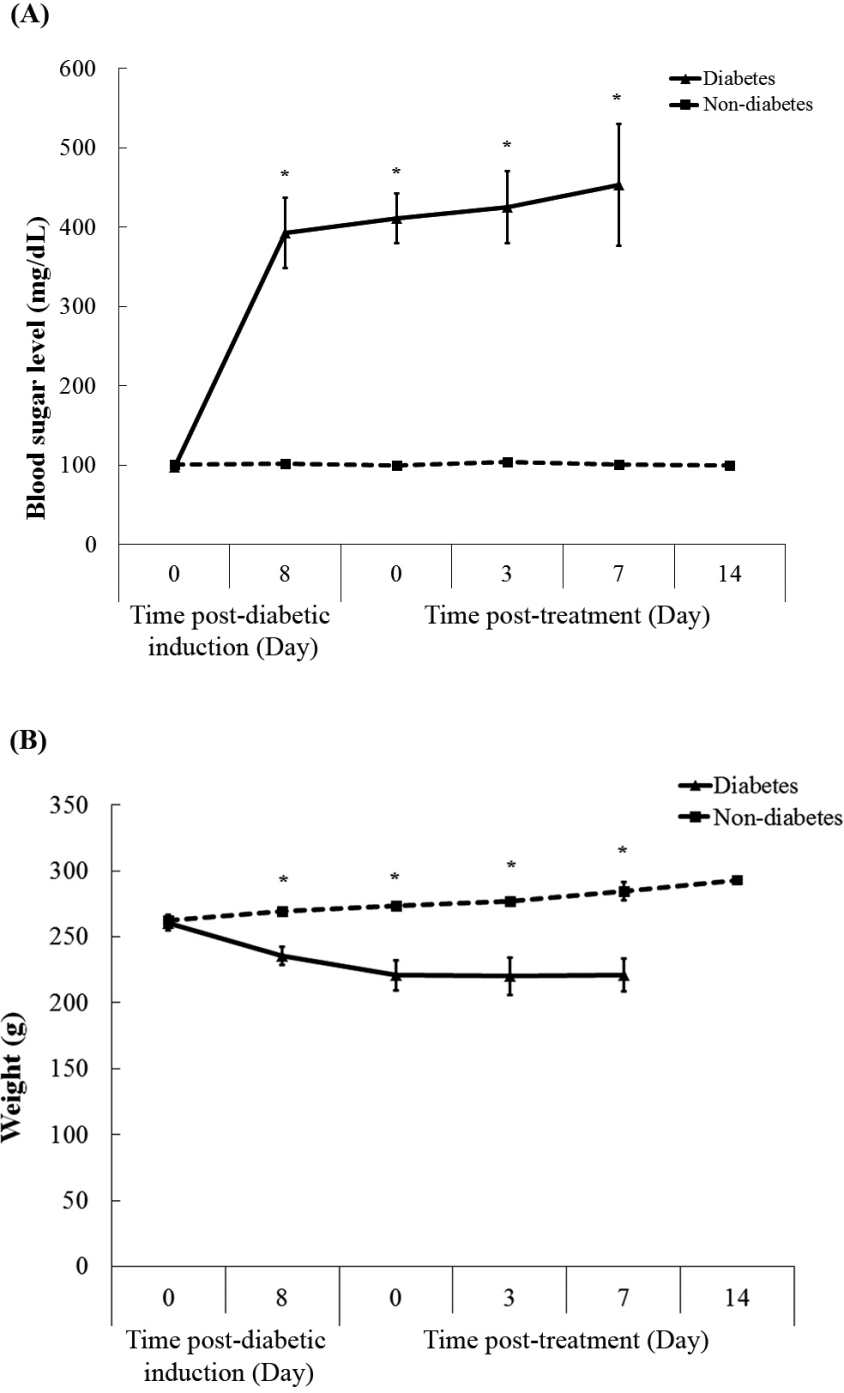
Blood sugar levels (A) and weight (B) of rats in both diabetic and non-diabetic groups. Data are expressed as mean ± standard error of measurement from animals (*n* = 8) per group. * *p* < 0.05.

Figure 2A shows blood sugar levels in diabetic and non-diabetic rats during the experiment. After seven days of injection, streptozotocin injection (50 mg/kg) was found to induce marked hyperglycaemia, compared with citrate-injected controls. Diabetic rats exhibited high blood sugar level at 398 mg/dL, while non-diabetic rats had their blood sugar levels less than the endpoint. Throughout the experimental period, high blood sugar levels in diabetic rats were observed, while those in non-diabetic groups remained stable approximately 106 mg/dL. Moreover, the clinical signs were presented in diabetic rats, including polyuria, polydipsia, and weight loss during the experiment (Fig. 2B).

### Rate of wound closure

**Figure 3:**
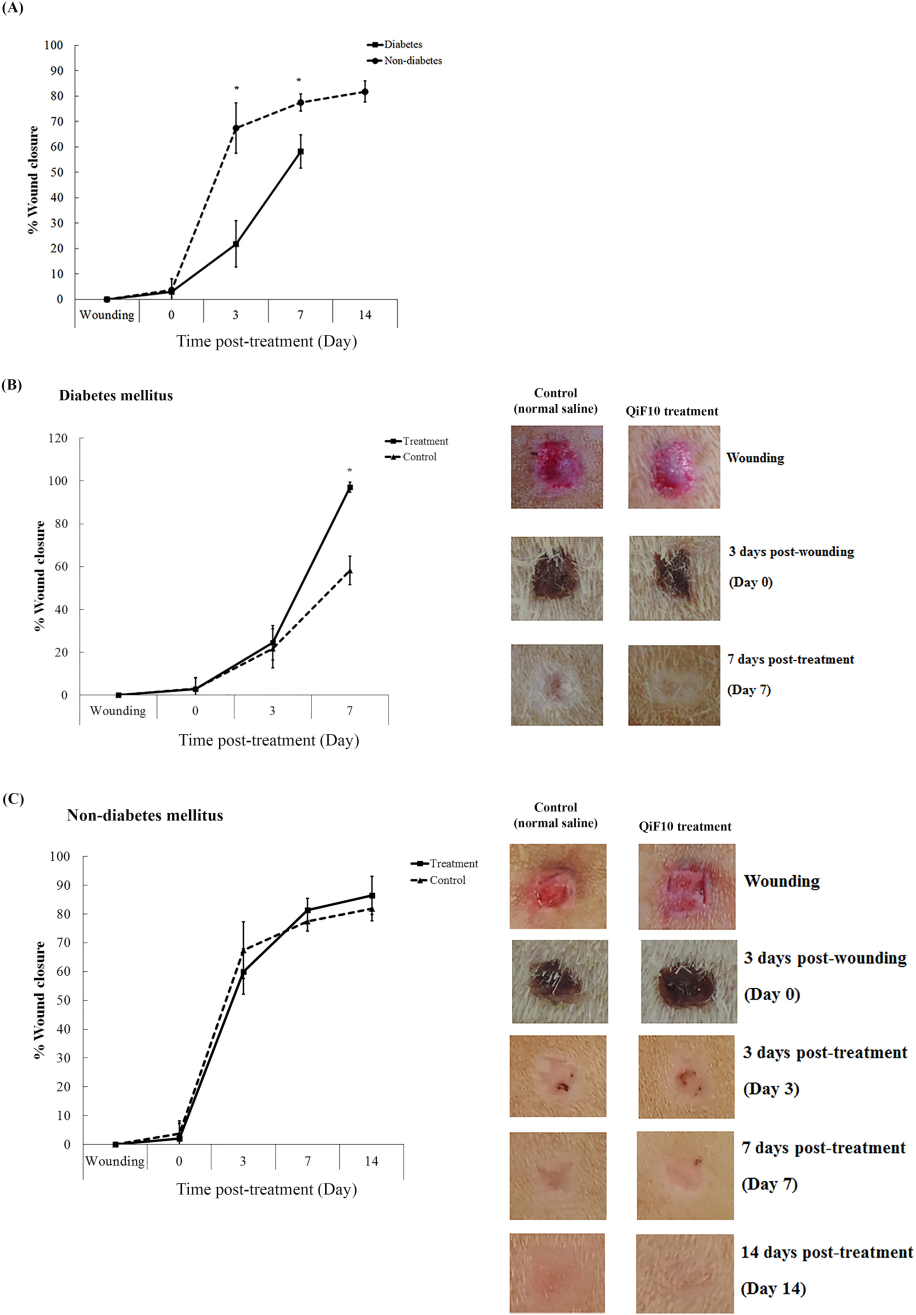
Wound healing response in diabetic and non-diabetic groups. Percentage wound closure and representative photographs. Comparison of rate of wound closure between untreated diabetic and non-diabetic rats (A). Wound healing activity in diabetic group (B) and non-diabetic group (C) after treatment with or without QiF10. Data are expressed as mean ± standard error of measurement from animals (*n* = 8) per group. * *p* < 0.05.

Figure 3 demonstrates wound healing activity in the treatment and the control groups of both diabetic and non-diabetic rats. After the wounds were left for three days, crust was observed in all groups. QiF10 and normal saline solution were initially applied on day 4 post-wounding in the treatment and the control groups, respectively. The rate of wound closure in diabetic rats was profoundly delayed throughout the experiment, compared with non-diabetic rats (Fig. 3A). Diabetic wounds treated with QiF10 appeared to heal faster than the saline-treated wounds (Fig. 3B). On day 3 after treatment, the wound area in both the treatment and the control groups was not significantly different (*p* < 0.05). In contrast, on day 7, the significant difference (*p* < 0.05) in percentage of wound closure in response to QiF10 treatment was observed with 94%, when compared with normal saline-treated wounds (50.18%).

In the non-diabetic group, percentage wound closure and wound characteristics between the treatment and the control groups were not different (Fig. 3C). On day 7, an increasing rate of wound closure was found in both the treatment and the control groups with 82.39% and 76.66%, respectively (Fig. 3C). By the end of the study, percentage wound closure in the treatment and the control group was noticed at 87% and 80%, respectively.

### QiF10 enhances wound healing process

**Figure 4:**
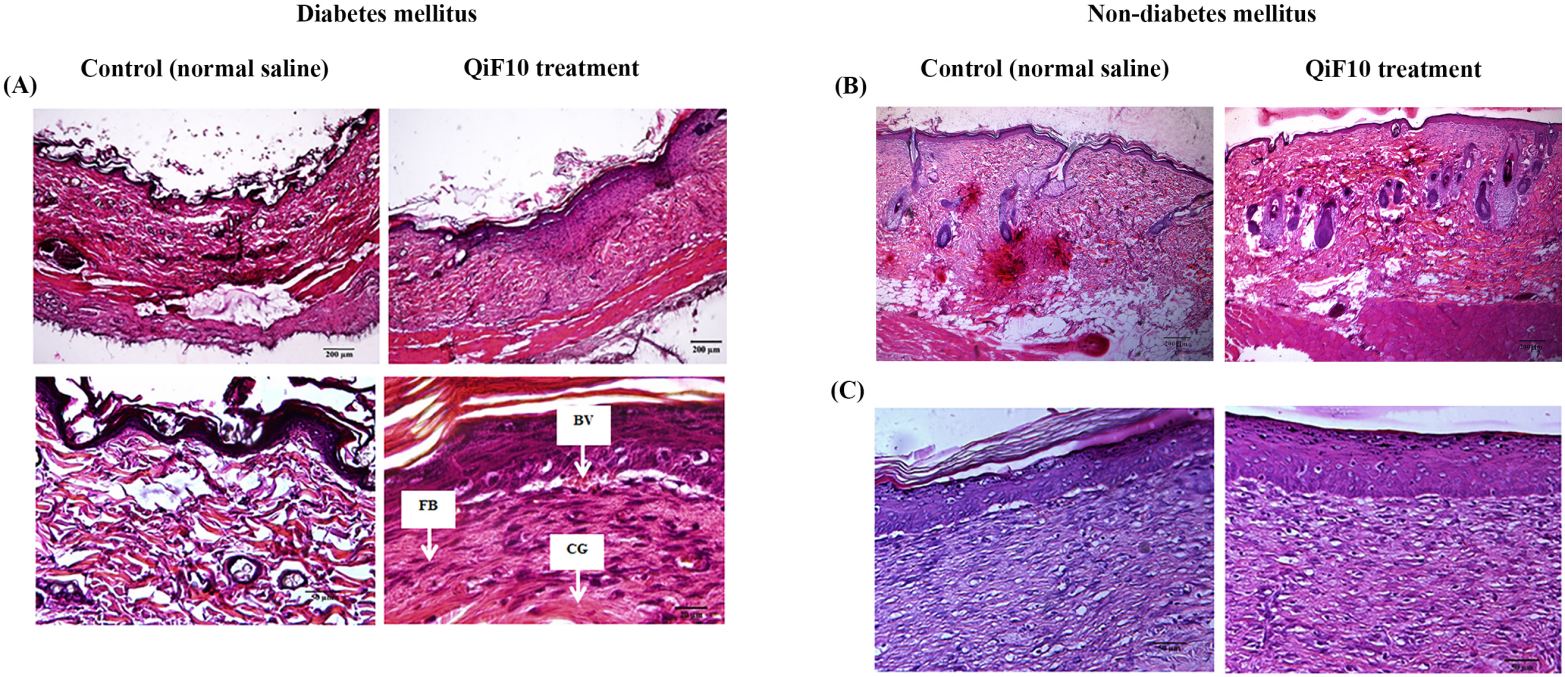
Histological images of diabetic (A) and non-diabetic wounds (B–C). On day 7, untreated diabetic wound tissues illustrated incomplete epithelialization and minimal cellular infiltration, whereas diabetic wounds treated with QiF10 demonstrated nearly complete re-epithelialization, large numbers of cell migration, especially fibroblast cells (FB) with collagen deposition (CG), and blood vessel (BV) at the wound area (A). In contrast, there were no obvious effects in QiF10 and normal saline treated non-diabetic wounds on day 7 (B) and day 14 (C).

To observe tissue changes, histological analysis was performed by staining with H&E. Fig. 4 illustrates the histological images of diabetic wounds and non-diabetic wounds treated with or without QiF10. Before initial treatment, incomplete epithelialization was observed with less number of cells and collagen deposition in all groups. Moreover, a long gap in wound area was presented in the dermis layers. After treatment for seven days, QiF10-treated diabetic wounds showed better re-epithelialization and collagen deposition than the control wounds (Fig. 4A). In addition, it has been developed abundant granulation tissue with a large number of cells containing fibroblast, collagen, capillaries, as well as inflammatory cells (Fig. 4A). In contrast, minimal cellular infiltration and collagen deposition were indicated in wounds treated with normal saline solution (Fig. 4A). Loosely packed collagen fibre and partial re-epithelialization were noted in normal saline treated wounds (Fig. 4A). This lack of cellular proliferation and granulation tissue formation led to delayed wound healing process.

Figures 4B–4C demonstrates histological images of non-diabetic wounds treated with or without QiF10. After seven days of the intervention, both QiF10 and normal saline-treated wounds were partially re-epithelialized (Fig. 4B). A complete re-epithelialization that contained granulation tissue rich in fibroblasts, collagen, and inflammatory cells in the dermis layers was shown in both groups by day 14 (Fig. 4C). Compared with diabetic wounds, non-diabetic ulcers had normal wound healing process with large numbers of cellular infiltration and collagen production (Figs. 4A, 4B). Moreover, regular differentiated keratinocyte cells were clearly observed in both the treatment and the control wounds from the non-diabetic group, resulting in rapid and complete re-epithelialization (Fig. 4C).

## Discussion

This study was focused on the development of a new topical therapeutic agent using nutgalls of *Quercus infectoria* for treatment of diabetic ulcers. QiF10, the most active best formulation based on antibacterial activity, demonstrated a remarkable ability to enhance wound healing process in diabetic rats. In contrast, no obvious effect was observed in non-diabetic wounds treated with QiF10, which may be due to their regular pattern in the wound healing process to rapidly repair themselves with a timely and orderly healing pathway (Velnar, Bailey & Smrkolj, 2009).

Wound healing, a normal complex process initiated after injury, is divided into four phases, including haemostasis, inflammation, proliferation, and tissue remodeling. The process requires coordinated interactions among various cell types, biochemical mediators, and extracellular matrix molecules within a specific time. Unlike acute wounds, diabetic ulcers show many molecular abnormalities in healing cascade involving defects in fibroblast and keratinocyte functions (Lerman et al., 2003; Lan et al., 2008), angiogenesis impairment (Loomans et al., 2004), and loss of phagocytic activity (Khanna et al., 2010). This study revealed that diabetic wounds displayed impairment in re-epithelialization, cell migration and proliferation, as well as granulation tissue formation, leading to deteriorated wound healing process. These abnormal patterns were clearly improved by QiF10 treatment. During proliferation phase, fibroblasts are stimulated to migrate and proliferate into the wound for production of the matrix proteins, hyaluronan, fibronectin, proteoglycans, as well as collagen fibre. Previous studies demonstrated that fibroblasts isolated from diabetic wounds had lower migratory activity and mitogenic responses, compared with non-diabetic wounds (Lerman et al., 2003; Xuan et al., 2014). These cellular abnormalities impede the formation of granulation tissues and extracellular matrix molecules, resulting in non-healing wounds. After QiF10 treatment, improvement of wound healing in diabetic wounds was observed with abundant fibroblast cells, accumulation of granulation tissues, and extracellular matrix formation around the wound area. One of the crucial stages for wound repairing is re-epithelialization which involves the activity of keratinocytes to move across the wound bed for restoration of epidermal layers. Due to high glucose levels, migration and proliferation of keratinocytes were impaired, resulting in inadequate re-epithelialization (Lan et al., 2008).

Diabetic wound infection is one of the main factors disturbing inflammatory response and delaying wound healing process. Most diabetic wounds are generally infected with complex microorganisms that are composed of both Gram positive and Gram negative bacteria. Our previous study noticed that the ethanolic extract of *Quercus infectoria* possessed very broad spectrum antibacterial activity against several wound pathogens, including *S. aureus*, MRSA, *Pseudomonas aeruginosa*, *Acinetobacter baumanii*, as well as *Escherichia coli* (Voravuthikunchai, Chusri & Suwalak, 2008). Moreover, the extract could reduce staphylococcal biofilm formation which is an important barrier to protect the bacteria from host immune system and antimicrobial treatment (Chusri, Phatthalung & Voravuthikunchai, 2012). Under diabetic condition, inflammation and oxidative stress have been extremely prolonged with a sustained expression of inflammatory cytokines, proteolytic enzymes, and large amounts of reactive oxygen species that directly damage protein structures of extracellular matrix components and modify the function of several cell types, leading to an impaired wound healing process (Mirza & Koh, 2015). A previous report confirmed that the nutgall extract has possess strong anti-inflammation properties by inhibition of several main inflammatory mediators, including histamine, serotonin, prostaglandin E2, and phorbol-12-myristate-13-acetate (Kaur et al., 2004). Very recently, nutgall extract manifested the ability to reduce diabetes-induced inflammation and oxidative stress through the suppression of Set7/NF-κB pathway in macrophages (Chokpaisarn et al., 2017). Moreover, the extract can act as an anti-oxidant agent to scavenge many deleterious free radicals, especially hydrogen peroxide and hydroxyl radical that can severely degrade wounded tissues (Kaur, Athar & Alam, 2008).

Gallic acid has been previously demonstrated as an active compound for anti-oxidant activity by up-regulation of antioxidant genes, superoxide dismutase 2, catalase, and glutathione peroxidase 1, in HaCaT cells (Yang et al., 2016). Furthermore, gallic acid significantly suppressed inflammation and attenuated DNA damage under hyperglycemic environment (Kuppan et al., 2010; Lee et al., 2015). In addition, it has been reported to stimulate cell migration of keratinocytes and fibroblasts cultured in both normal and high glucose conditions (Yang et al., 2016). Therefore, the activity of QiF10 for promotion of wound healing process in diabetic rats may be resulted from biological properties of gallic acid which is the main component presented in the extract (Kaur, Athar & Alam, 2008). Wounds treated with QiF10 improved due to homeostasis of inflammatory cells, oxidative reaction and cellular function improvement.

## Conclusions

QiF10 had the ability to enhance the wound healing process of diabetic ulcers by promoting cell proliferation, re-epithelialization, and granulation tissue formation. Therefore, it could be used as a novel alternative treatment for diabetic wounds.

##  Supplemental Information

10.7717/peerj.3608/supp-1 Data S1 Raw data for ABTS assay

10.7717/peerj.3608/supp-2 Data S2 Raw data for DPPH assay

10.7717/peerj.3608/supp-3 Data S3 Raw data for total flavonoid determination

10.7717/peerj.3608/supp-4 Data S4 Raw data for total phenolic determination

10.7717/peerj.3608/supp-5 Data S5 Raw data for *in vivo* experiment

10.7717/peerj.3608/supp-6 Table S1 Stability of QiF10 after 3 freeze-thaw cycles

## Additional Information and Declarations

### Data Availability

The following information was supplied regarding data availability: The raw data is supplied in the Supplemental Files.
