# Supplementary material for: Potential wound healing activity of Quercus infectoria formulation in diabetic rats
Source: PeerJ. 2017 Jul 24;5:e3608. doi: 10.7717/peerj.3608 (PMC5527956; doi:10.7717/peerj.3608)
Supplement: Table S1 [file peerj-05-3608-s006.docx]

Table Stability of QiF10 after 3 freeze-thaw cycles.

Cycles Temperature Characteristics of the formulation

Colour Precipitation Phase separation pH

Before Dark brown - - 4-5

1 4 ºC Dark brown - - 4-5

60 ºC Dark brown - - 4-5

2 4 ºC Dark brown - - 4-5

60 ºC Dark brown - - 4-5

3 4 ºC Dark brown - - 4-5

60 ºC Dark brown - - 4-5
